# Evaluation of knowledge graph embedding approaches for drug-drug interaction prediction in realistic settings

**DOI:** 10.1186/s12859-019-3284-5

**Published:** 2019-12-18

**Authors:** Remzi Celebi, Huseyin Uyar, Erkan Yasar, Ozgur Gumus, Oguz Dikenelli, Michel Dumontier

**Affiliations:** 1 Institute of Data Science, Maastricht University, Maastricht, 6200 Netherlands; 2 Computer Engineering Department, Ege University, Izmir, 35100 Turkey

**Keywords:** Drug-drug interaction, Paired data, Disjoint cross-validation, Realistic evaluation

## Abstract

**Background:**

Current approaches to identifying drug-drug interactions (DDIs), include safety studies during drug development and post-marketing surveillance after approval, offer important opportunities to identify potential safety issues, but are unable to provide complete set of all possible DDIs. Thus, the drug discovery researchers and healthcare professionals might not be fully aware of potentially dangerous DDIs. Predicting potential drug-drug interaction helps reduce unanticipated drug interactions and drug development costs and optimizes the drug design process. Methods for prediction of DDIs have the tendency to report high accuracy but still have little impact on translational research due to systematic biases induced by networked/paired data. In this work, we aimed to present realistic evaluation settings to predict DDIs using knowledge graph embeddings. We propose a simple disjoint cross-validation scheme to evaluate drug-drug interaction predictions for the scenarios where the drugs have no known DDIs.

**Results:**

We designed different evaluation settings to accurately assess the performance for predicting DDIs. The settings for disjoint cross-validation produced lower performance scores, as expected, but still were good at predicting the drug interactions. We have applied Logistic Regression, Naive Bayes and Random Forest on DrugBank knowledge graph with the 10-fold traditional cross validation using RDF2Vec, TransE and TransD. RDF2Vec with Skip-Gram generally surpasses other embedding methods. We also tested RDF2Vec on various drug knowledge graphs such as DrugBank, PharmGKB and KEGG to predict unknown drug-drug interactions. The performance was not enhanced significantly when an integrated knowledge graph including these three datasets was used.

**Conclusion:**

We showed that the knowledge embeddings are powerful predictors and comparable to current state-of-the-art methods for inferring new DDIs. We addressed the evaluation biases by introducing drug-wise and pairwise disjoint test classes. Although the performance scores for drug-wise and pairwise disjoint seem to be low, the results can be considered to be realistic in predicting the interactions for drugs with limited interaction information.

## Background

Adverse Drug Events (ADEs) are a significant threat to public health. A study by Lazarou et al. [1] estimates 6.7% of hospitalized patients experience serious adverse drug effects with fatality rate 0.32% in the USA. In 2014, 807,270 cases of serious ADEs were reported in the United States, resulting in 123,927 lost lives [2]. ADEs present a financial burden to the healthcare system due to the costs of further hospitalization, morbidity, mortality, and health-care utilization. Drug-drug interactions (DDIs), which occasionally arise through co-prescription of a drug with other drug(s), may cause an undesired effect other than its principal pharmacological action [3]. A significant number of adverse drug effects (approximately 3-26%) leading to hospital admission are attributed to unintended drug-drug interactions (DDIs) [4]. Patient groups such as elderly patients and cancer patients are more likely to take multiple drugs simultaneously, which increases their risk of DDIs [5, 6]. Current approaches to identifying DDIs, include safety studies during drug development and post-marketing surveillance after approval, offer important opportunities to identify potential safety issues, but are unable to provide complete set of all possible DDIs [7]. Thus, the drug discovery researchers and healthcare professionals might not be fully aware of potentially dangerous DDIs. Predicting potential drug-drug interaction helps reduce unanticipated drug interactions and drug development costs and optimizes the drug design process. Thus, there is clear need for automated methods for predicting DDIs.

In recent years, biological data and knowledge bases have been increasingly built on Semantic Web technologies and knowledge graphs are used for information retrieval, data integration, and federation [8]. Many bioinformatics databases have begun to present their data as Linked Open Data (LOD), a graph data model, using Semantic Web technologies [9, 10]. The knowledge graphs provide a powerful model for defining the data, in addition to making it possible to use underlying graph structure for extraction of meaningful information.

Researchers have used features based on the properties such as targets, side effects, fingerprint (a bit-vector describing chemical structure) and indications for prediction of drug-drug interactions [11–14]. These features are either incorporated into a large sparse binary vector or a dense similarity vector which has few dimensions. Neither representation is ideal for machine learning tasks, and they both entail effort-intensive feature engineering. In recent years, several approaches have been proposed to generate features automatically from LOD[15]. Approaches such as FeGeLOD [16] and RapidMiner Linked Open Data Extension [17] have used different unsupervised feature generation strategies to enrich data with the features obtained from LOD. Yet, efficient feature representation can be learned using the knowledge graph embedding approaches in which the nodes/edges are mapped to low-dimensional dense vectors [18]. The representation of the drugs can be learned by graph embedding approaches in a purely unsupervised and task-independent way, which would provide informative, independent and discriminative features to predict potential DDIs. It is also possible to use these feature vectors in other downstream tasks such as drug-target, drug-adverse effect prediction. Moreover, knowledge graph embeddings can be used to make predictions for the drugs that have no interaction information. Owing to LOD, the presence of an entity (drug) is sufficient to enable embedding vectors for machine learning to be extracted. Most drugs and hence DDIs could be included in the training set with this intention. Similarity-based approaches, in contrast, do not allow for the calculation of various similarities for many drugs due to lack of drug information. Besides, graph embedding approaches using only one type of interaction data (homogeneous graph) such as node2vec [19], DeepWalk [20] cannot make predictions for those drugs with no interaction.

In this work, we have extended our previous work [21] by applying realistic evaluation cross-validation (CV) schemes on different knowledge graph embedding predictors using DrugBank [22, 23], KEGG [24] and PharmGKB [25] knowledge graphs to predict potential DDIs. The results show that performance of drug vector representation which was used to train classifiers is comparable to the existing pharmacological similarity methods for DDI prediction. The AUC score of 0.93 and F-Score of 0.86 were achieved based on ten cross-validations with the vector representations of drugs for the DrugBank dataset.

When developing a new drug, the researchers are asked to predict possible interactions of new chemical entity (potential drug) with approved drugs but often there is little information available related to that chemical entity. Moreover, the researchers in the computational drug discovery field do not use realistic settings to evaluate their predictions, instead preferring to use traditional CV, which leads to optimistic results. The traditional CV where test pairs might share components with training pairs is prone to over-fitting due to systematic biases in networked/paired data [26–29]. There have been a few studies which have addressed this issue [27, 28, 30, 31]. Some studies [30, 31] demonstrated how well their methods perform to make predictions for new drugs which lacked interaction data. These studies only consider the case where new drugs and their interactions in the test set were hidden from training set. Park et al. [27] proposed a more systematical approach, in which he divided the reference data into 3 classes to evaluate the protein-protein interaction prediction methods more realistically. These are C1, C2 and C3; C1, in which test pairs share both proteins with the training set; C2, in which test pairs share only one protein with the training set; and C3, in which test pairs share neither protein with the training set. However, the setting is rather complex and the failure to provide an algorithm or code make it challenging to reproduce the setting. Guney also suggested similar cross-validation settings for DDI prediction: non-disjoint, disjoint and pairwise disjoint CV [28, 32]. Non-disjoint CV is the same as the traditional CV, while disjoint and pairwise disjoint CV are similar to C2 and C3 scenarios, respectively. In disjoint CV, the data set is partitioned into k-groups such that each group contains the DDIs where one of the drugs can appear only in that group and cannot appear in other groups. In pairwise-disjoint CV, the data set is partitioned such that each group contains the DDIs where both of the drugs can appear only in that group and cannot appear in other groups. In his disjoint cross validation setting, however, the partitioning of the pairs into groups is done according to the first component of the pair. Simply grouping according to the second component would produce different sampling of the data set and thus might lead to inconsistency across folds.

Here, we propose a simple disjoint CV scheme adapted from [27–29] to evaluate DDI predictions for the cold-start drugs which have no DDI information known in the training set. One advantage of the proposed approach over Guney’s approach is to produce consistent sampling of data across folds. Another advantage is to use of single training data for both test cases in each fold, thus reducing computational time. We designed two scenarios: (i) for the prediction of interactions of cold-start drugs with existing drugs (drug-wise disjoint CV) and (ii) for the prediction of interactions when both drugs in a pair were cold-start drugs (pairwise disjoint CV). Recently in [33], authors proposed cross-validation schemes (CV1, CV2) where CV1 is used to assess the prediction that new drugs interact with known drugs, while CV2 is used to assess the prediction that new drugs interact with new drugs. While CV1 is the same as our drug-wise CV, CV2 combines two kinds of sampling; within-group and between-group. Within-group sampling contains DDI pairs between only a set of drugs that is left for testing while between-group sampling contains DDI pairs between two different sets of drugs that are left for testing. Our approach handles two different scenarios (CV1 and CV2-within group) and produce one training set and two test sets for one fold. Ours share the exact same training set in one fold for both CVs. However, CV2 produces combinations of two samplings (within-group and between-group) which might produce different number of rounds/samplings (eg. for 10-K CV, 10 rounds (within-group) and 45 rounds (between-group)). Averaging the results of these two different samplings might create a bias since the number of rounds and the ratio of training set and test set could be hugely different. Shi et al. [29] proposes a similar CV approach where their first scenario (S1) corresponds to traditional CV and second scenario (S2) corresponds drug-wise CV and third scenario (S3) is pairwise CV. However, they did not provide any formal definition and efficient algorithm for their CV.

Our contribution can be summarized as follows : i) comparison of different knowledge graph embedding approaches on DDI prediction task ii) evaluation of different knowledge graphs as background knowledge for feature learning iii) testing DDI prediction task for the disjoint CV scenarios.

## Related work

Researchers have used various approaches and data sources to predict novel drug interactions [7]. These approaches include extracting DDI statements from medical texts and drug event reports [34], inferring DDI mechanism [35] by integrating knowledge from several sources and using network proximities [36]. Previous studies regarding prediction of DDIs have tried to summarize the related works under various taxonomic classifications such as similarity-based and classification-based, similarity-based and feature-based [12, 30]. These taxonomic classifications do not sufficiently explain the distinctions between approaches. We classify the studies under memory-based and model-based approaches on basis of the taxonomic classification of the Recommender Systems [30, 37]. The memory-based approach relies on loading similarity scores into memory and recommending directly (most similar neighbors) based on this data. With the model-based approach, a model is derived from data and a recommendation is yielded by this model.

Memory-based approaches predict a candidate drug pair based on its most similar known drug pairs. Finding well-known interacting drugs that are very similar to a drug pair provides evidence to support an interaction between these candidate drugs. Some of these methods are described below:

Ferdousi et al. [38] used carriers, transporters, enzymes and targets (CTET) from the DrugBank database to predict DDIs. In this study, 2189 approved drugs, 45,530 known drug interactions, and 2,349,236 unknown drug pairs were investigated. To determine DDIs, they collected all CTETs associated with each drug and formed binary vectors. They then aimed to identify DDIs by applying many similarity methods to these combined vectors. They subsequently predicted more than 250,000 potential new DDIs using inner product-based similarity measures (IPSMs) from these similarity methods. To train the final classifier, they used 2004 features.

Vilar et al. [13] developed a method based on the molecular structural similarities of drugs. In this study, 928 drugs and 9454 DDI were collected from DrugBank v3.0, from which the interactions were used as a reference data. The drug-drug similarity was created through a combination of DrugBank DDIs and molecular fingerprint modeling. The similarity of the drug pairs was calculated based on the Tanimoto coefficient and molecular fingerprints.

Shi et al. [39] proposes a matrix factorization model to predict enhancive (positive) and degressive (negative) drug drug interactions (DDIs) using drug binding proteins as a feature. They try to find balanced/unbalanced drug communities on the network of enhancing and depressive DDIs and predict DDIs for the cold-start scenario.

The most commonly used features for model-based approaches were pharmacological similarities [40]. Gottlieb et al. [11], by using different drug similarity metrics, developed a new prediction framework called INDI. INDI trained a logistic classifier using 7 similarities, also using them to calculate their maximum likelihood by using known DDIs. Cheng et al. [14] presented the HNAI framework for predicting drug interactions using phenotypic, therapeutic, structural, and genomic similarities of drugs. Cami et al. [36] have trained a logistic classifier by extracting the pharmacological and graph/network qualities between drugs. Zhang et al. [41] used a label propagation method on drug chemical infrastructure, drug side effect and drug off-side effects. Li et al. [42] have developed a Bayesian network that combines drug molecular similarity and drug phenotypic (side effect) similarity to predict the combination effect of drugs. Zhang et al. [12] collects a variety of drug data and thus predicts DDIs by integrating chemical, biological, phenotypic and network data. The work by Shi et al. [43] is focused on predicting synergistic drug combination rather than drug-drug interactions using only positive relationships with one-class SVM. In [44], the authors integrate four drug features, chemical substructures, targets, enzymes and pathways, by mapping drugs in different feature spaces into the common interaction space through sparse feature learning. Then, the linear neighborhood regularization is used to describe drug–drug relations in the interaction space by using known drug–drug interactions.

There are also other works which use feature vectors as input to machine learning methods. Luo et al. [45] proposed a 611 feature vector method based on molecular structure. Later, the logistic model was trained with these feature vectors to find potential DDIs for 2515 drug molecules.

Abdelaziz et al. [30] presented Tiresias, a similarity-based framework for predicting DDIs. They used 1014 features derived from pharmacological similarities and from drug text and similarity based on the knowledge graph embeddings (TransH and HolE). Each feature represents the similarity value of the known interacting drug pair to the most similar drug pair. An integrated knowledge base consisting of DrugBank, UMLS, DailyMed, Uniprot and CTD datasets was created as an RDF data network and this integrated information network includes entities such as enzymes, chemical structures and pathways, drug properties and relationships. This knowledge graph was used to calculate the global similarity measure between drugs. Precision, recall, F-score and AUPR were used as evaluation criteria.

Hameed et al. [46] presented a Positive-Unlabeled Learning (PUL) approach based on the Growing Self Organizing Map (GSOM) cluster to estimate the potential negative data required for binary classification methods for DDI inference. They predicted that 589 drug pairs from 6036 DDIs obtained from DrugBank did not interact with each other, considering these as a negative class in the binary classification method. The proposed approach which used the 5-cross validation, produced Precision of 0.97, Sensitivity of 0.98 and F1-Score of 0.97.

## Methods

**Fig. 1:**
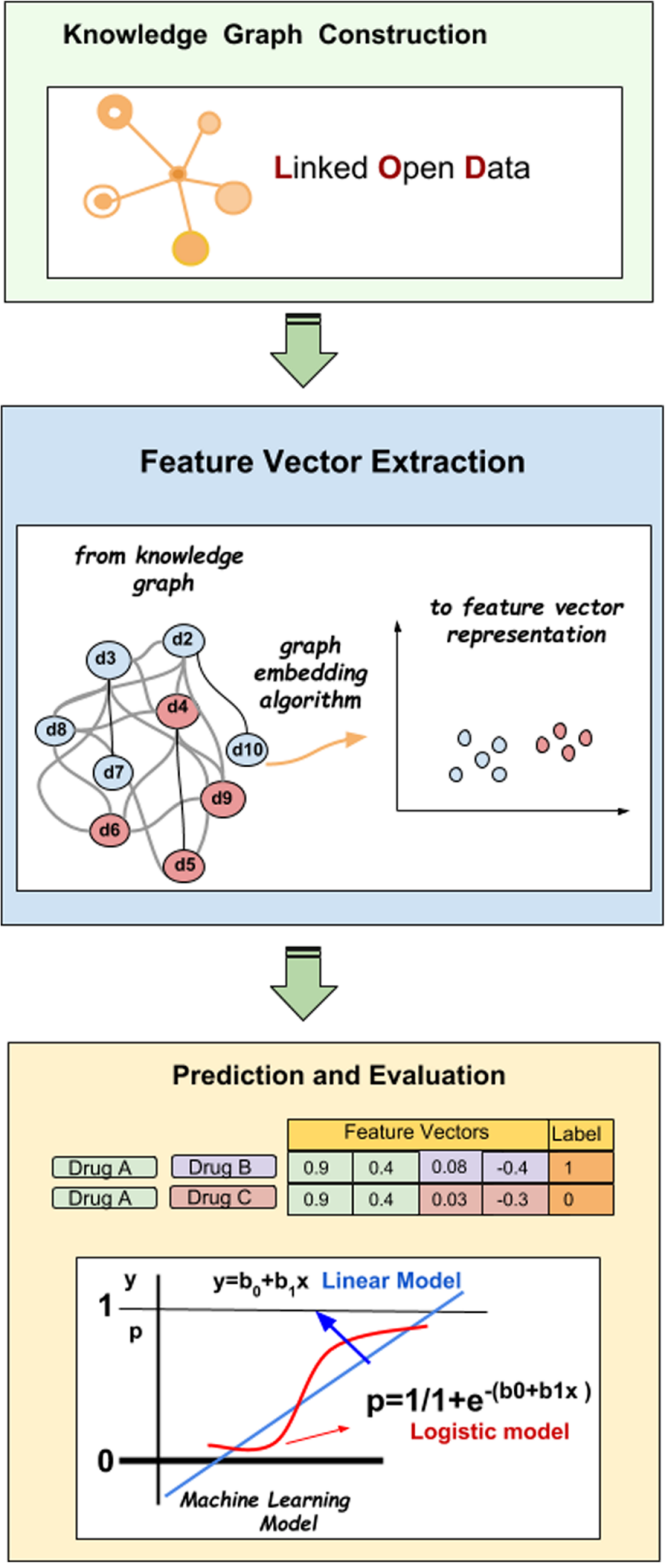
Overview of our methodology

**Fig. 2:**
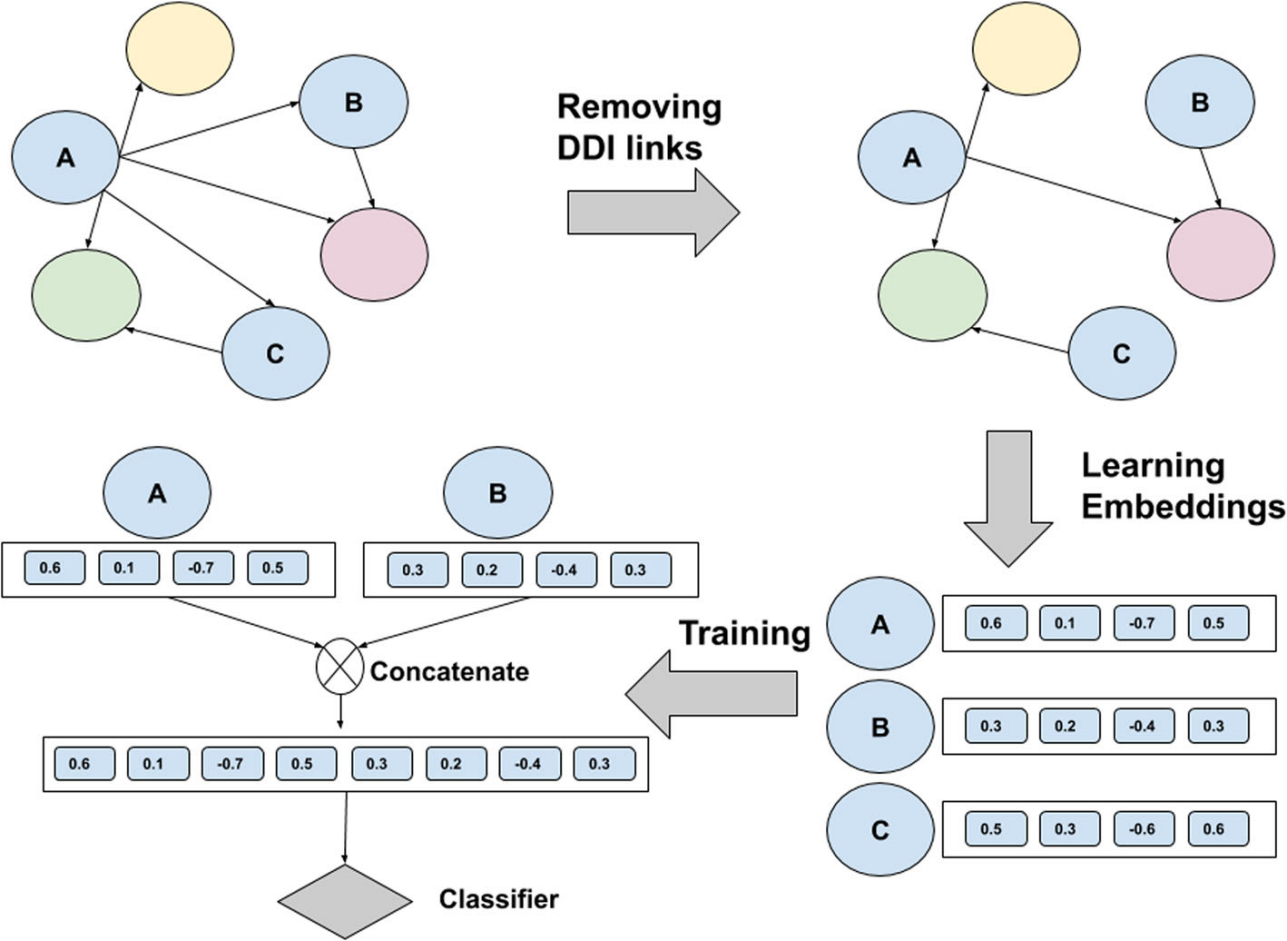
A toy example on how to apply Knowledge Graph Embedding to DDI prediction. A, B and C nodes represent drug entities

The steps of our RDF Graph Embedding based DDI prediction methodology are shown in Fig. 1. The first step is to construct knowledge graph data in RDF format. And then as second step, the feature vector of drugs is extracted using the knowledge graph by applying different Graph Embedding approaches namely RDF2Vec [47], TransE [48] and TransD [49]. Note that, graph embedding approaches such as Node2Vec, DeepWalk and LINE can be applied for homogeneous graphs but can not be used for knowledge graphs that contain multiple entity and relation types. For that reason, we did not use these embeddings for our evaluation. The last step is to predict drug interactions using extracted feature vectors by applying three different classifiers: Logistic Regression, Naive Bayes and Random Forest. We provide a toy example of the drug knowledge graph and the workflow on how to apply Knowledge Graph Embedding to model DDI prediction in Fig. 2.

### Knowledge graph construction

Linked Open Data (LOD) is a technique for publishing, describing, and linking data [50]. Linked open data is a potential source of background knowledge for modeling predictive machine learning and building content-based recommender systems [47]. LOD is used to identify resources with Uniform Resource Identifiers (URI) [51] and through standards such as the RDF (Resource Description Framework) [52] which is a powerful data model to describe and exchange resources on the Web.

We used an already linked open biological dataset, called Bio2RDF [53], as background knowledge to extract drug features. Bio2RDF is an open-source project that integrates numerous Life Sciences databases available on different websites, providing a data integration service for scientific researchers. Bio2RDF creates a large RDF graph that interlinks data from major biological databases related to biological entities such as drug, protein, pathway and disease. In this study, DrugBank, KEGG and PharmGKB datasets within Bio2RDF project release 4.0 were used as the background knowledge graph. We removed DDI links from these knowledge graphs to eliminate bias on the prediction task. The number of triples, entities and relation types related to each dataset are given in Table 1.

**Table 1:** The number of triples, entities and relation types related to each knowledge sources to be used for embedding learning

Dataset	# of triples	# of entities	# of relation types
DrugBank	2,588,933	574,152	76
KEGG	14,362,294	4,362,395	301
PharmGKB	2,976,202	830,433	78
DrugBank + KEGG + PharmGKB	19,806,314	5,645,847	445

### Feature vector extraction

We have tested multiple successful approaches proposed for knowledge graph embeddings to generate features from graphs such as RDF2Vec, TransE and TransD. These approaches are explained in detail in the following subsections.

#### RDF2Vec

RDF2Vec is a recently published methodology that adapts the language modeling approach of word2vec [54] to RDF Graph Embeddings. Word2vec trains a neural network model to learn vector representation of words, called word embeddings. It maps each word to a vector of latent numerical values in which semantically and syntactically closer words will appear closer in the vector space. The hypothesis which underlies this approach is that closer words in word sequence are statistically more dependent. RDF2Vec applies a similar approach on RDF graphs, considering the entities and relations between entities by converting the graph into set of sequences (walks or paths) and training the neural network model to learn vector representation of entities from the RDF graph.

- **Random Graph Walks**
  *G*(V,E) is a graph with *V* set of nodes and *E* set of edges. The random walk algorithm was used to generate *P*_*v*_ paths at depth d starting at each vertex v in V. At first iteration, the algorithm traverses the direct outgoing edges of a root vertex (vr), then randomly exploring the connected edges through visited vertices until d iterations is reached. The union of all the *P*_*vr*_ walks, starting from all entities (vr) in the knowledge network were used as a set of sequences to train artificial neural network models.
- **Neural Network Training**
  Each word (entity) is trained to maximize its log probability according to the context words within the fixed-size window. Each word in the vocabulary is represented by two vectors; input and output vectors. While learning the input vectors is cheap, learning the output vectors is very expensive. Approximation techniques such as hierarchical softmax and negative sampling have been developed for efficient training. Word2vec introduces two architectures to obtain vector embedding representation of words: Continuous Bag-of-Words (CBOW) and Skip-Gram (SG).
  **Continuous Bag-of-Words Model**
  The CBOW model is a two-layer artificial neural network model that predicts a target word using context words in near proximity. Given word sequence *w*_1_,*w*_2_,*w*_3_,..,*w*_*T*_, CBOW tries to maximize the average log probability of the target word as follows:
  1 $$ \frac{1}{T}\sum\limits_{t=1}^{T} log(p(w_{t}| w_{t-c} + \cdots + w_{t+c}))  $$
  where *c* is the context window and p defined as :
  2 $$ p(w_{t}| w_{t-c} + \cdots + w_{t+c}) = \frac{exp\left({\overline{v}}^{T} {{v^{\prime}}_{w_{t}}}\right)}{ \sum\nolimits_{w=1}^{V} exp\left({\overline{v}}^{T} {{v^{\prime}}_{w}}\right)}  $$
  where *v*^′^_*w*_ is output vector of word *w*, *V* is the complete vocabulary of words and $\overline {v}$ is the averaged input vector of all the context words.
  **Skip-Gram Model**
  While CBOW predicts the word given the context, the Skip-gram predicts the context of the given word. It tries to find useful word representations to predict the words around the target word in a training document or sentences. Given word sequence *w*_1_,*w*_2_,*w*_3_,..,*w*_*T*_ and context window size *c*, Skip-gram maximizes the average log probability as follows:
  3 $$ \frac{1}{T}\sum\limits_{t=1}^{T} \sum\limits_{-c\leq j \leq c, j=0}^{} log(p(w_{t+j}| w_{t}))  $$
  where *p* is defined using softmax function as follows:
  4 $$ p(w_{t+j}| w_{i}) = \frac{exp\left({{v^{\prime}}^{T}_{w_{t+j}}} {v_{w_{t}}}\right)}{ \sum\nolimits_{w=1}^{V} exp\left({{v^{\prime}}^{T}_{w_{k}}} {v_{w_{t}}}\right)}  $$
  where *v*_*w*_ and *v*^′^_*w*_ are the input and the output vector of the word w, and *V* is the complete vocabulary of words.

#### TransE

**Fig. 3:**
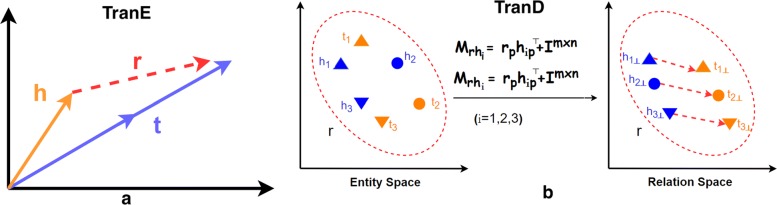
Illustrations of Translation-based embeddings; (**a**) TransE and (**b**) TransD embedding method

TransE uses an energy-based model to embed the knowledge graph into low-dimensional vector space. In TransE, the relations in a knowledge graph are represented as translation from head entity to tail entity so that vector embeddings should satisfy *h*+*r*≈*t* where a triple (h,r,t) in training set *S* is composed of two entities h,t∈E (the set of entities) and relationship *r*∈*R* (the set of relationships). A vector representation of every entity and relation in the knowledge graph could be computed by learning a neural network model, which minimizes energy function *f*(h,r,t)=∥*h*+*r*−*t*∥, vector norm of difference between head entity plus relation and tail entity in embedding space (see Fig. 3a). For a triple (h,r,t) in knowledge graph, the embedding head h would be close to the embedding tail t by adding the embedding relation r whereas for any corrupted triple (*h*^′^,r,t^′^) not in knowledge graph, that would be opposite. TransE will minimize the energy function as follows:

5 $$ \mathcal{L}=\sum\limits_{(h,r,t)\in S}\sum\limits_{(h^{\prime},r,t^{\prime})\in S^{\prime}_{(h,r,t)}}\left[\gamma+d(h+r,t)-d(h^{\prime}+r,t^{\prime})\right]_{+}  $$

where [*x*]_+_ is positive part of x, *γ*>0 is hyperparameter and *d* is dissimilarity measure that can be defined as distance in *L*_1_ or *L*_2_-norm. *S*^′^ is the set of corrupted triples defined as

6 $$ S^{\prime}_{(h,r,t)}=\left\{(h^{\prime},r,t)\vert h^{\prime}\in E\right\}\bigcup\left\{(h,r,t^{\prime})\vert t^{\prime}\in E\right\}  $$

Since entity and relation embedding vectors lie on the same space, TransE is convenient for modeling one-to-one relations, but is insufficient for one-to-many, many-to-one and many-to-many relations.

#### TransD

In TransD, each entity or relation is defined by two vectors; one being the embedding vector of an entity or a relation, the other the projection vector. The projection vector represents the way to project an entity vector into a relation vector space to be used to construct mapping matrices. In Fig. 3b, each cluster represents an entity pair appearing in a triplet of relation *r*. *M*_rh_ and *M*_rt_ are mapping matrices of *h* and *t*, respectively. *h*_ip_,*t*_ip_(*i*=1,2,3) and *r*_*p*_ are projection vectors. *h*_*i*⊥_ and *t*_*i*⊥_(*i*=1,2,3) are projected vectors of entities. The projected vectors should satisfy *h*_*i*⊥_+*r*≈*t*_*i*⊥_(*i*=1,2,3). Every entity-relation pair has a unique mapping matrix. Thus, it can handle one-to-many, many-to-one and many-to-many relations. In addition, TransD has no matrix-by-vector operations which can be replaced by vectors operations.

### Prediction and evaluation

#### Reference DDI datasets

We used various reference DDI data sets to train and evaluate our classifiers on embedding features for the DDI task:

- **DrugBank v4:** This is an online database that contains detailed drug information such as drug structure, pathway, pharmacodynamic and pharmacokinetic effects of the drug and interaction data [23]. We have obtained interaction data as of February 2015 which contains 1514 drugs and 96,942 DDIs.
- **DrugBank v5:** This is the latest major version of DrugBank database as of July 2018. It has various enhancements over early versions [22] and contains 288,856 distinct pairwise DDIs spanning 2551 drugs.
- **KEGG:** KEGG extracted DDIs from the interaction tables of Japanese product labels. We obtained 26,664 DDIs where drugs were mapped to DrugBank IDs via the work done by [55].

#### Evaluation

In a traditional k-fold CV setting for machine learning task, the samples are partitioned into equal sized k-subsets in which one subset is used as a test set and the remaining data is used to train the model for a fold. The results of folds are averaged to make a more accurate estimate of model prediction performance. However, when samples are in the form of a pair of objects, the traditional CV leads to optimistic results due to the presence of the same objects in both the training set and the test set [27]. To make realistic evaluation of DDI prediction task, we propose two scenarios similar to what Park [27] and Guney [28] suggested for the paired-input methods: (i) drug-wise disjoint CV and (ii) pairwise disjoint CV. To create these scenarios, the drugs that form the drug pairs are split into 2 clusters: cold-start and existing drugs. The term cold-start drugs implies that these drugs have no known DDIs in the training set and the term existing drugs implies the drugs have DDIs in the training set. Let Drugs_coldstart_ denote the set of hidden drugs for which no DDIs are known at training. The rest of the drugs will be called existing drugs denoted by Drugs_existing_. The set of known interacting drugs in reference data, *KDDI*, will be partitioned into three subsets; KDDI_train_,KDDI_drugwise_ and KDDI_pairwise_ such as

7 $$\begin{array}{*{20}l} KDDI_{{train}} &= \{(d_{1},d_{2})| d_{1} \in Drugs_{{existing}} \land d_{2} \in Drugs_{{existing}} \} \end{array} $$

8 $$\begin{array}{*{20}l} KDDI_{{drugwise}} &= \{(d_{1},d_{2})| (d_{1} \in Drugs_{{existing}} \land d_{2} \in Drugs_{{coldstart}}) \\ &\quad\lor (d_{1} \in Drugs_{{coldstart}} \land d_{2} \in Drugs_{{existing}}) \} \end{array} $$

9 $$\begin{array}{*{20}l} KDDI_{{pairwise}} &= \{(d_{1},d_{2})| d_{1} \in Drugs_{{coldstart}} \land d_{2} \in Drugs_{{coldstart}} \} \end{array} $$

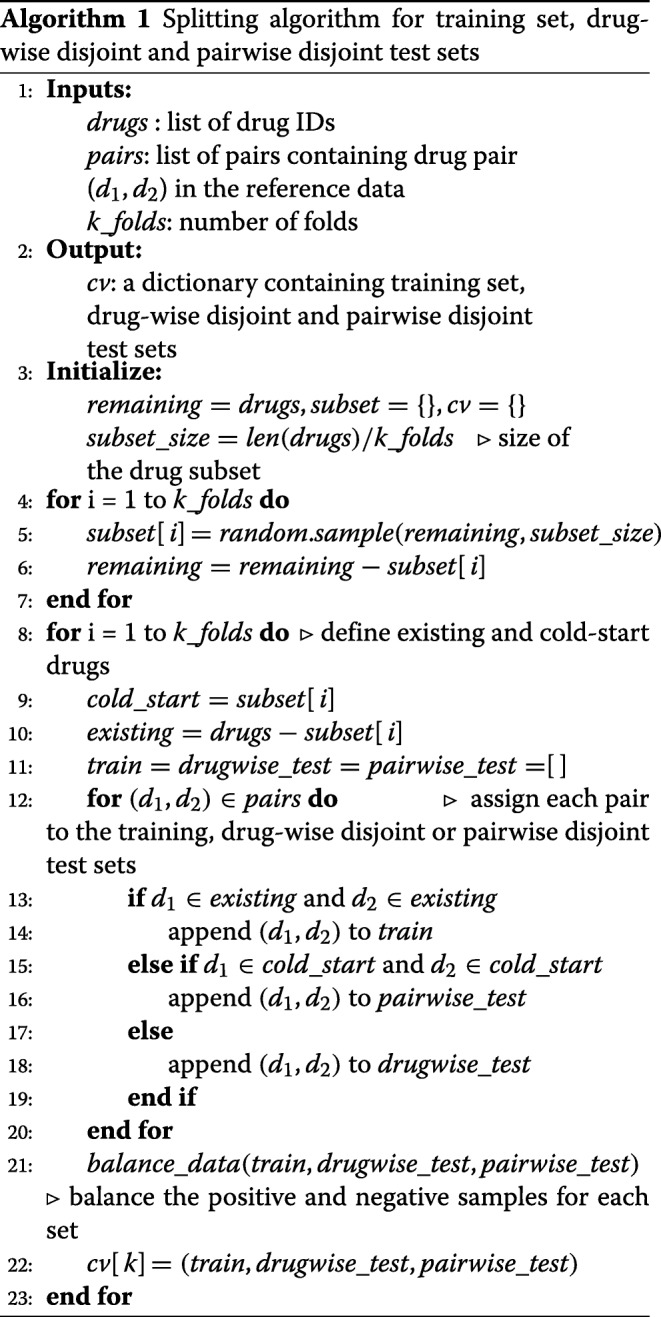


**Fig. 4:**
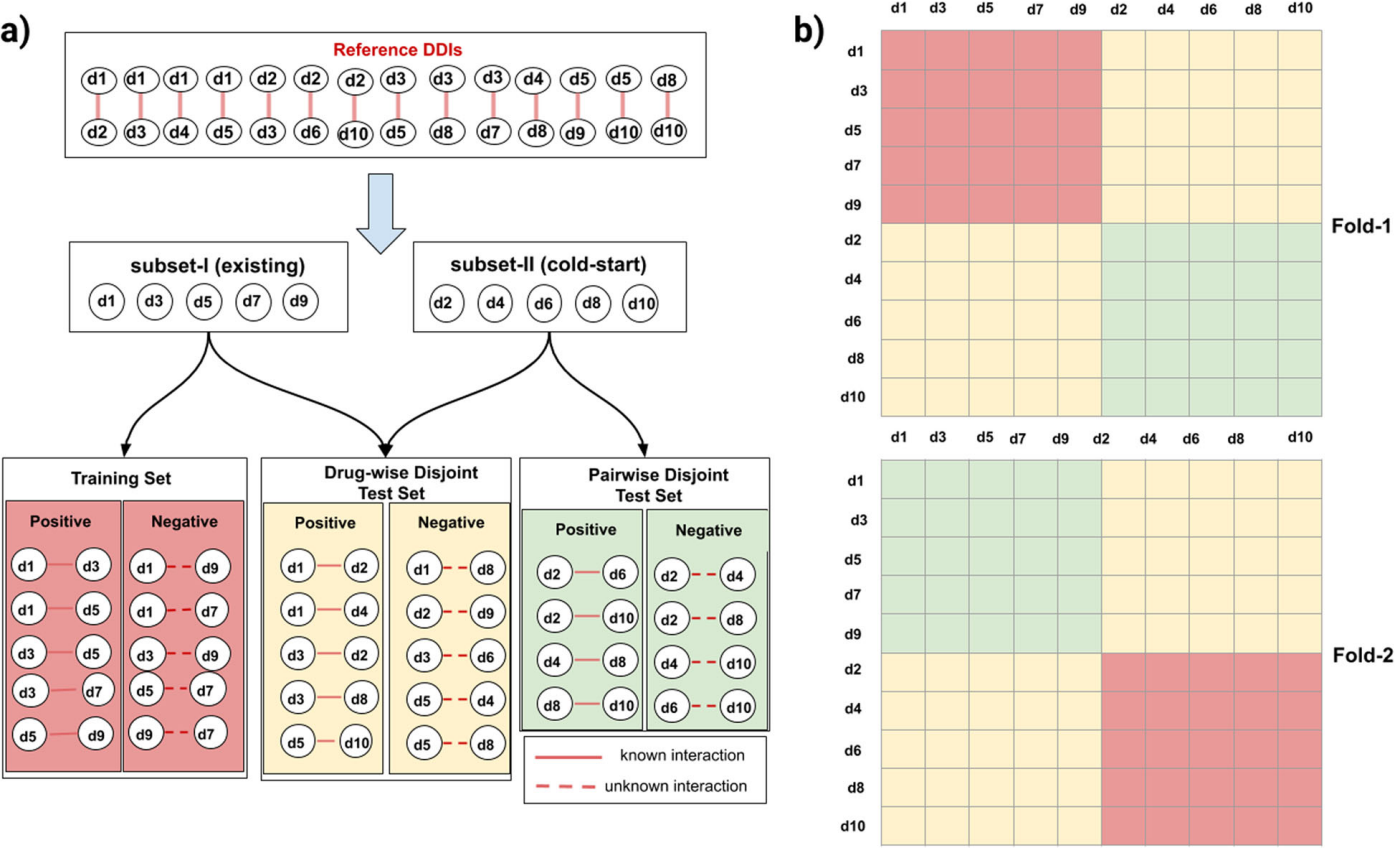
To illustrate a partitioning for 2-fold cross validation, we consider a toy example of DDI prediction, in which the reference data has 10 drugs and 14 DDIs. **a** The train-test generation workflow for a fold. Drugs are randomly split into 2 groups where one group is used as cold-start drugs to generate test sets, while the remaining drugs (existing) are used to generate a training set for a fold. Splitting the drugs into 2 groups leads to partitioning of drug pairs into 3 sets: training set, drug-wise disjoint test set and pairwise disjoint test set. The pairs which include both components from existing drugs are assigned to the training set. The interactions between existing drugs and cold-start drugs are assigned to the drug-wise disjoint test set and likewise, the interactions between cold-start drugs are assigned to the pairwise disjoint test set. In other words, the drug-wise test set will contain the drug pairs each of which shares only one element with training set. The pairwise test set will contain the drug pairs in which neither component is shared with the training set. **b** Partitioning of the drug-drug pair space into training and test subsets for each fold. The pair space is represented by a table with 10×10 cells. The drug-drug pair space is divided into different blocks, which account for training, drug-wise testing and pairwise testing parts, and are filled with red, yellow and green colors respectively

The Algorithm 1 constructs training and test sets for a given reference DDIs. It inputs the interactions and drugs in the reference data and the number of folds for cross-validation and outputs the three datasets; training, drug-wise and pairwise test sets for each fold. Instead of dividing the drug pairs into equal size k-groups, we split the drugs into k-groups where the drugs in one group will be considered as cold-start drug set and the remaining (k-1) groups will be used as existing drug set for each fold. In Fig. 4, we give an example of 2-fold CV partitioning.

In addition, we performed time-slice CV in which an earlier version of the dataset is used as the training set and the new version that is created at a later point in time is used as the test set. Crichton and colleagues[56] have stated that a time-slice setting where predictors are evaluated on how well they predict chronologically later links would be a realistic setting. However, this setting requires multiple snapshots of data for the evaluation to be performed.

Let *D*_*t*1_={(*x*_*i*_,*y*_*i*_)|i=1 to m} denote the first snapshot of data for training at time *t*_1_ with *m* data points. And let $D_{t_{2}} = \{ (x_{i}, y_{i}) | \mathrm {i = 1 \ to \ n}\}$ denote the second snapshot of data at time *t*_2_ with *n* data points. Note that, *t*_2_>*t*_1_ in terms of time. So, for our time-slice CV, after having trained our classification model with $D_{t_{1}}$, the difference between the two datasets $D_{t_{2}} - D_{t_{1}}$ (e.g. novel interactions) is used as a test set. In order to apply 10-fold CV, we randomly split the test set to 10 groups and averaged the obtained scores for every group.

#### Data balance

For DDI prediction using supervised machine learning, a binary classifier needs negative and positive example sets. In previous studies the negative set typically was chosen randomly from unknown interactions. Alternatively, the set of all unknown interactions could be designated as the negative set, but designating all unknown interactions as the negative set creates a data balance issue, influencing performance metrics (such as AUPR and F1-score). Other studies accounted for this issue through a random undersampling from these unknown interactions at a ratio corresponding to the positive set [14], or inferring negatives by clustering [46]. In this study, the negative samples were taken randomly from unknown drug pairs in sample size equivalent to the positive samples.

#### Evaluation metrics

While many studies use the AUC score in computational prediction for DDIs, some studies such as [30, 46] have emphasized that this score is insufficiently accurate, therefore metrics such as AUPR and F1 score are viable alternatives. We used evaluation metrics including F1 score, AUC and AUPR to accurately measure the performance of our classifiers.

#### Machine learning models

We used three well known classes of machine learning models Logistic Regression, Naive Bayes and Random Forest to train our classifiers using Scikit-learn machine learning package. The parameters used for building the classifiers are as follows; C =0.01 for Logistic Regression, Gaussian version for Naive Bayes and number of estimators = 200 and max depth = 8 for Random Forest. The parameters were tuned according to 10-fold traditional CV.

#### Embedding parameters

We generated walks to be used as input to RDF2Vec where the graph walk parameters are depth = 1,2,3,4 and walks per entity = 250. And we trained the word2vec model using CBOW and SG neural network architectures with the following parameters; window size = 5, number of iterations = 5, negative samples = 25 and dimension = 100. The size of each drug vector is 100. We opted for embedding parameter values that were used in the study [47]. We conducted several experiments with different parameter values but didn’t observe any significant change. To represent feature vector of a drug pair, we concatenated embedding vectors of each drug in the pair. Thus, the classifiers used 200 features for prediction of DDIs. The default parameters given by OpenKE (openke.thunlp.org) were used for TransE and TransD models.

## Results

We first performed the experiments applying Logistic Regression, Naive Bayes and Random Forest on DrugBank knowledge graph with different CV using three well known knowledge graph embedding methods, namely RDF2Vec, TransE and TransD. The results of the experiments on traditional CV, drug-wise and pairwise disjoint CV, and time-slice CV are shown in Table 2. The scores shown in the result tables are the means of ten runs of each experimental setting. We used DrugBank v5 as reference data using 10-fold CV for traditional and disjoint settings and were able to extract features for 2124 drugs of these 2551, filtering out the drugs that have no calculated feature vector. Thus, the number of DDIs was reduced to 253,449. For time-slice scheme, DrugBank v4 was used to train the classifiers and new interactions added to DrugBank v5 were predicted using the classifiers trained on embedding features. RDF2Vec methods (Skip-Gram and CBOW) have significantly outperformed the other graph embedding methods. RDF2Vec embedding vectors with Skip-Gram mostly achieved the best performance values. The best AUC, AUPR and F1-score values obtained are 0.93, 0.92 and 0.85 respectively using Random Forest learning algorithm with traditional CV. For the disjoint cases, the scores tended to drop in all machine learning models compared to traditional CV. Although the Random Forest was the best model for most of cases, we observed Logistic Regression and Naive Bayes would produce better F-scores in pairwise disjoint and time-slice settings.

**Table 2:** Evaluation of different embedding methods in various CV schemes

		Traditional CV				Drug-wise CV				Pairwise CV				Time-slice CV		
Embedding	ML Model	AUPR	F1	AUC		AUPR	F1	AUC		AUPR	F1	AUC		AUPR	F1	AUC
RDF2Vec	Logistic Regression	0.78	0.71	0.78		0.76	0.69	0.76		0.73	0.66	0.74		0.75	0.68	0.76
CBOW	Naive Bayes	0.68	0.63	0.70		0.68	0.63	0.70		0.68	0.63	0.70		0.71	0.67	0.73
	Random Forest	**0.92**	**0.85**	0.92		0.79	0.69	0.78		0.75	0.64	0.74		**0.80**	0.69	**0.80**
RDF2Vec	Logistic Regression	0.79	0.72	0.79		0.77	0.70	0.77		0.75	**0.68**	**0.75**		0.76	0.69	0.76
SG	Naive Bayes	0.76	0.68	0.74		0.75	0.68	0.74		0.75	0.67	0.73		0.78	**0.72**	0.78
	Random Forest	**0.92**	**0.85**	**0.93**		**0.81**	**0.71**	**0.80**		**0.76**	0.63	**0.75**		**0.80**	0.68	**0.80**
TransE	Logistic Regression	0.78	0.70	0.76		0.73	0.67	0.73		0.72	0.67	0.72		0.75	0.68	0.76
	Naive Bayes	0.75	0.69	0.73		0.72	0.68	0.71		0.72	**0.68**	0.71		0.76	**0.72**	0.76
	Random Forest	0.90	0.83	0.91		0.76	0.69	0.77		0.73	0.64	0.73		0.77	0.65	0.78
TransD	Logistic Regression	0.74	0.68	0.74		0.74	0.67	0.74		0.72	0.66	0.72		0.74	0.70	0.75
	Naive Bayes	0.72	0.68	0.71		0.72	0.67	0.71		0.72	0.67	0.71		0.73	0.70	0.73
	Random Forest	0.91	0.84	0.91		0.77	0.69	0.77		0.73	0.64	0.73		0.78	0.68	0.78

Bio2RDF DrugBank knowledge graph and DDIs from DrugBank v5 were used in the evaluation. We considered these CV settings: traditional CV, disjoint CV (drug-wise, pairwise) and time-slice CV. The settings are explained in the Evaluation section. (Bold: best score)

**Fig. 5:**
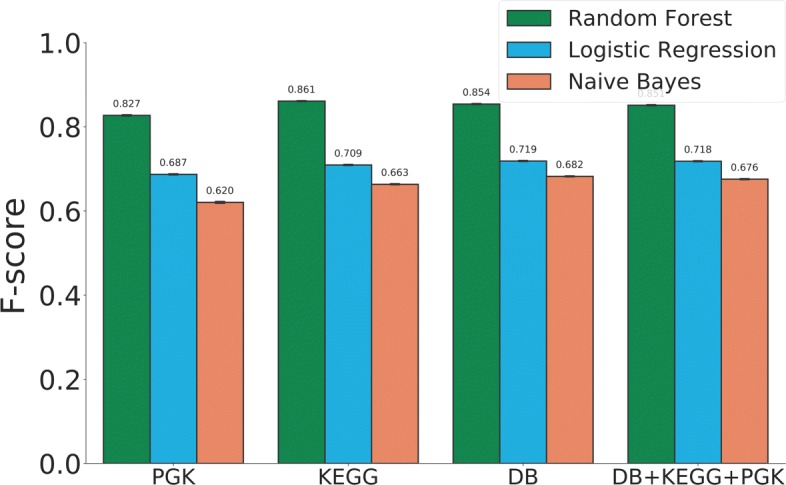
Comparison of the knowledge graph sources in predicting DDIs by F-scores for traditional CV

We next used different knowledge graph sources to better understand the effect of the learned embedding vectors on prediction performance using DrugBank v5. Figure 5 shows the F-scores of DDI prediction task when different knowledge sources were used to learn embedding feature vectors using the best embedding approach, RDF2Vec with Skip-Gram. We obtained an F-score of 0.85 for DrugBank, 0.82 for PHARMGKB, 0.86 for KEGG. Thus, the PHARMGKB and the KEGG data sources, when used alone, showed no significant improvement on prediction performance compared to DrugBank. In addition, when these multiple data sources are used together the predictive power of drug vectors did not improve significantly. We conclude that DrugBank knowledge graph was sufficient alone to characterize the drugs for DDI prediction task, instead of using integration of multiple knowledge graphs.

**Fig. 6:**
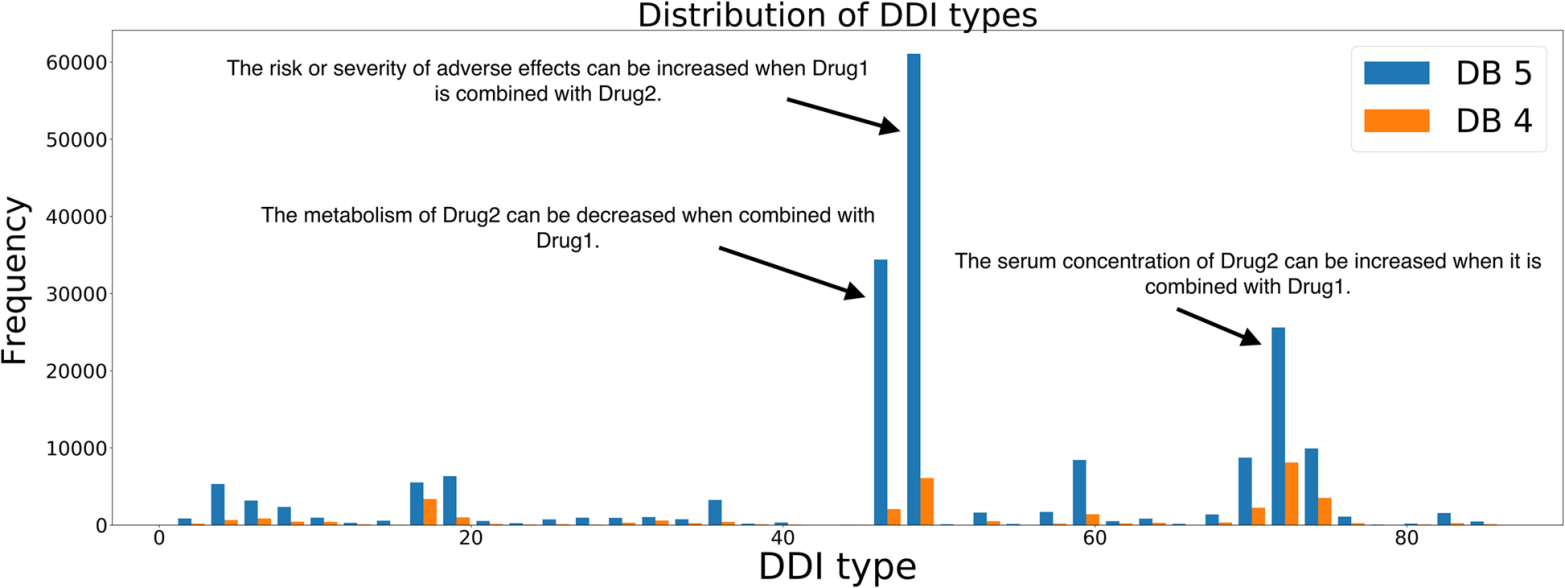
Frequency distribution of DrugBank 5 and DrugBank 4 in terms of different DDI types

**Fig. 7:**
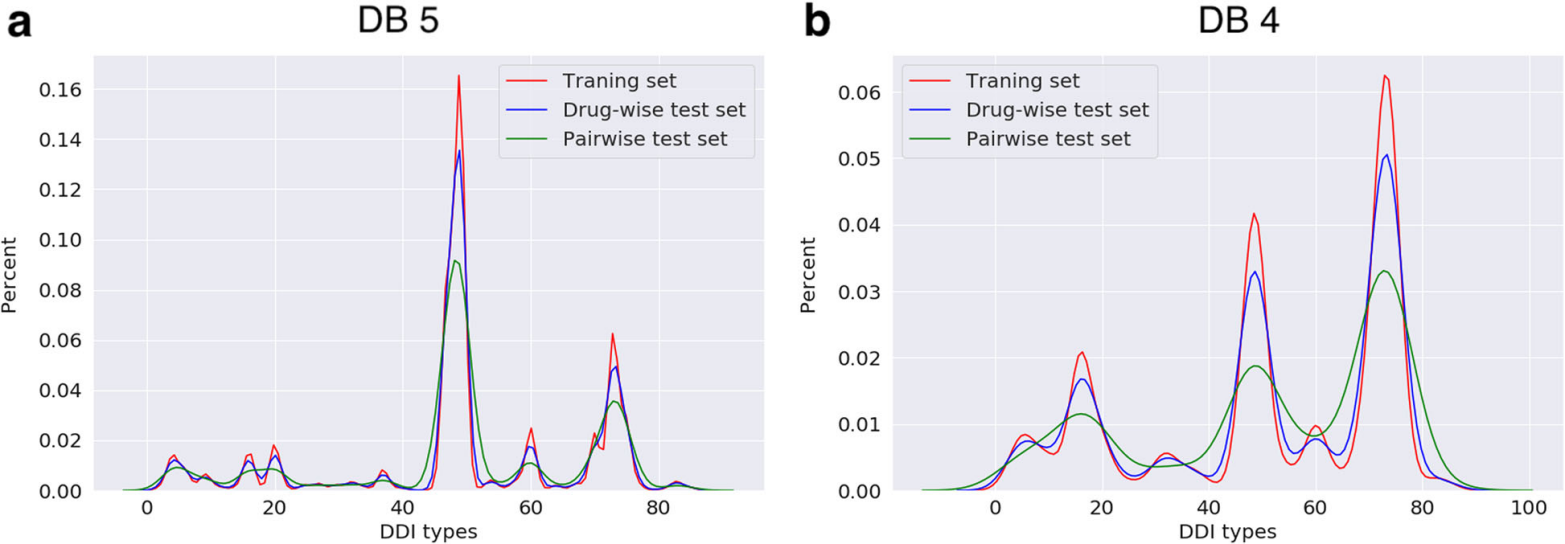
Mean percent distribution of DDI types of training and test sets of (**a**) DrugBank 5 and (**b**) DrugBank 4 using proposed 10-fold disjoint cross-validation method

To show how proposed CV divides the dataset by DDI types, we compared the distribution of training and test sets produced by our CV method in terms of DDI types. For this purpose, we have used 86 DDI types for DrugBank 5 dataset obtained from the DeepDDI study [57]. The DDI types were assigned by classifying the description sentences into general sentence structures. These general sentence structures provide information about pharmacokinetic or pharmacodynamic mechanisms. Using these types, we plotted the frequency distribution of DDIs observed in DrugBank 5 and DrugBank 4. Figure 6 shows distribution of these interactions. 60% of the 192284 DDIs in DrugBank 5 were assigned to 3 DDI types. We examined the distribution of the training set and the test sets to see whether the test samples follow a distribution similar to that of the training samples in terms of DDI types. We compared distributions between the test set and training set generated using 10-fold disjoint cross validation where the number of cold-start drugs was determined to be 10% of the total number of drugs. We observed that the percent distribution of DDI types observed in the test sets produced by our CV is consistent with those of the training using Pearson’s chi-squared test (*p*>0.05 for all folds, accepting null hypothesis. See Supplementary Table 1, Additional file 1). In Fig. 7, the mean percent distribution of DDI types of ten folds using DrugBank 5 and DrugBank 4 was shown.

**Comparison with the state-of-art methods:** In spite of the high number of methods which have been proposed for DDI prediction, their results have had insufficient basis for comparison because of the differing terms of their reference data (known DDIs) and evaluation methodologies of the studies. We provide the prediction performance of RDF2Vec Skip-Gram embedding vectors on the previous benchmark data sets, DrugBank v4 and KEGG, that were used by other studies in Table 3. The Tiresias framework [30], which is one of the most noteworthy studies, uses both pharmacological similarities and similarities from embedding features using DrugBank v4. Tiresias has reported an F-score of 0.85 and AUPR of 0.92, all features included, as their best results and an F-score of 0.81 and AUPR of 0.89 with only pharmacological similarity features (equivalent performance with INDI [11]). Our embedding using the DrugBank v4 with similar settings achieved a high F-score of 0.86 and AUPR of 0.92. It shows that the proposed embedding based method is comparable to current state-of-the-art methods for traditional CV.

**Table 3:** Performance values for different CV schemes using DrugBank v4 and KEGG as reference DDIs

		Traditional CV				Drug-wise CV				Pairwise CV		
Reference Data	ML Method	AUPR	F1	AUC		AUPR	F1	AUC		AUPR	F1	AUC
DrugBank 4	Logistic Regression	0.75	0.70	0.76		0.70	0.65	0.72		0.66	0.60	0.67
	Naive Bayes	0.71	0.66	0.72		0.70	0.65	0.70		0.68	**0.63**	0.69
	Random Forest	**0.90**	**0.84**	**0.91**		**0.78**	**0.69**	**0.79**		**0.69**	0.52	**0.70**
KEGG	Logistic Regression	0.77	0.71	0.78		0.69	0.63	0.70		0.62	0.54	0.62
	Naive Bayes	0.73	0.67	0.74		0.71	0.63	0.71		0.66	**0.58**	**0.67**
	Random Forest	**0.85**	**0.79**	**0.86**		**0.78**	**0.68**	**0.79**		**0.67**	0.38	**0.67**

The embedding generated by RDF2Vec with SkipGram was used in the experiments. (Bold: best score)

## Discussion

Using traditional CV strategies for paired/networked data creates a bias due to the inherent relations between paired samples, pointing to the potential danger in using them for evaluation. We propose a realistic CV scheme which considers two disjoint scenarios for DDI prediction: (i) drug-wise disjoint CV and (ii) pairwise disjoint CV. We have shown that a better realistic evaluation can be attained using the drug-wise disjoint scenario when drugs have limited interaction information. In fact, the drug-wise disjoint produced similar results with a time-slice setting, which is considered a more realistic scenario than traditional CV. Furthermore, in some cases, it may be desirable to predict the likelihood of interactions between newly developed drugs without interaction information. The pairwise disjoint CV is more appropriate for this case, even though the pairwise disjoint case can be considered the worst case scenario in evaluating the performances of the model for DDI.

We provided an empirical evaluation of our proposed CV for paired data and an algorithm to split paired data into train and test subsets, while also emphasizing its differences with the other CV methods proposed in the literature. Examining this issue from a network theoretic perspective (e.g. scale-freeness) will be an important step towards a more comprehensive assessment of the methods. In addition, we designed experiments to check whether the proposed CV method introduced additional bias when it split data into training and test sets, and found no evidence of bias. In this paper, we focused on comparing different embeddings for realistic CV settings. The study did not focus on other aspects of the evaluation such as negative sampling. A limitation of graph embeddings is that they are unable to provide the mechanistic explanations for predicted potential DDIs, given that the embedding predictors were constructed using a black box model (neural network model). However, knowledge graphs can provide interpretable outcome for a drug pair via mining rules or the paths of relationships of inferred interacting drug pairs.

## Conclusions

In this study, we showed that the knowledge embeddings are powerful predictors and comparable to current state-of-the-art methods for inferring new DDIs. Knowledge graph embedding approaches enabled us to extract features for a large number of drugs. Previous studies used much lesser known DDI samples (≈40−50*K*).

The presence of the same objects in both the training set and the test set produced flaws in the evaluation for paired data. We addressed the evaluation biases by introducing drug-wise and pairwise disjoint test classes. Although the performance scores for drug-wise and pairwise disjoint seem to be low, the results can be considered to be realistic in predicting the interactions for drugs with limited interaction information. We also consider temporal evaluation (referred as time-slicing) setting which takes the temporal evolution of the interaction graph into account and how well the links formed later could be predicted. But this evaluation setting might not be applicable for every datasets when there exist no multiple snapshots. Therefore, the proposed disjoint evaluation scheme would be better choice for paired inputs with no temporal data.

## Supplementary information

**Additional file 1** The frequency distribution of DDIs observed in training and test sets for each fold in 10-fold disjoint cross-validation and Chi-square test results for each fold, the file is in XLSX format.

## Data Availability

The datasets generated and/or analysed during this study along with the code for our models and instructions for their use are available under open licenses at https://github.com/rcelebi/GraphEmbedding4DDI/.

## References

[1] Lazarou J, Pomeranz BH, Corey PN (1998). Incidence of adverse drug reactions in hospitalized patients. JAMA.

[2] FDA. FAERS Reporting by Patient Outcomes by Year. 2018. https://www.fda.gov/Drugs/GuidanceComplianceRegulatoryInformation/Surveillance/AdverseDrugEffects/ucm070461.htm. Accessed 27 June 2018.

[3] Abubakar AR, Chedi BA, Mohammed KG, Haque M (2015). Drug interaction and its implication in clinical practice and personalized medicine. National J Physiol Pharm Pharmacol.

[4] Dechanont S, Maphanta S, Butthum B, Kongkaew C (2014). Hospital admissions/visits associated with drug–drug interactions: a systematic review and meta-analysis. Pharmacoepidemiol Drug Saf.

[5] Riechelmann RP, Tannock IF, Wang L, Saad ED, Taback NA, Krzyzanowska MK (2007). Potential drug interactions and duplicate prescriptions among cancer patients. J Natl Cancer Inst.

[6] Doubova Dubova SV, Reyes-Morales H, Torres-Arreola LdP, Suárez-Ortega M (2007). Potential drug-drug and drug-disease interactions in prescriptions for ambulatory patients over 50 years of age in family medicine clinics in mexico city. BMC Health Serv Res.

[7] Percha B, Altman RB (2013). Informatics confronts drug–drug interactions. Trends Pharmacol Sci.

[8] Katayama T, Wilkinson MD, Aoki-Kinoshita KF, Kawashima S, Yamamoto Y, Yamaguchi A, Okamoto S, Kawano S, Kim J-D, Wang Y, Wu H, Kano Y, Ono H, Bono H, Kocbek S, Aerts J, Akune Y, Antezana E, Arakawa K, Aranda B, Baran J, Bolleman J, Bonnal RJ, Buttigieg PL, Campbell MP, Chen Y-A, Chiba H, Cock PJ, Cohen KB, Constantin A, Duck G, Dumontier M, Fujisawa T, Fujiwara T, Goto N, Hoehndorf R, Igarashi Y, Itaya H, Ito M, Iwasaki W, Kalaš M, Katoda T, Kim T, Kokubu A, Komiyama Y, Kotera M, Laibe C, Lapp H, Lütteke T, Marshall MS, Mori T, Mori H, Morita M, Murakami K, Nakao M, Narimatsu H, Nishide H, Nishimura Y, Nystrom-Persson J, Ogishima S, Okamura Y, Okuda S, Oshita K, Packer NH, Prins P, Ranzinger R, Rocca-Serra P, Sansone S, Sawaki H, Shin S. -H., Splendiani A, Strozzi F, Tadaka S, Toukach P, Uchiyama I, Umezaki M, Vos R, Whetzel PL, Yamada I, Yamasaki C, Yamashita R, York WS, Zmasek CM, Kawamoto S, Takagi T (2014). BioHackathon series in 2011 and 2012: penetration of ontology and linked data in life science domains. J Biomed Semant.

[9] Smith B, Ceusters W, Klagges B, Köhler J, Kumar A, Lomax J, Mungall C, Neuhaus F, Rector AL, Rosse C (2005). Relations in biomedical ontologies. Genome Biol.

[10] Jupp S, Malone J, Bolleman J, Brandizi M, Davies M, Garcia L, Gaulton A, Gehant S, Laibe C, Redaschi N, Wimalaratne SM, Martin M, Le Novère N, Parkinson H, Birney E, Jenkinson AM (2014). The EBI RDF platform: linked open data for the life sciences. Bioinformatics.

[11] Gottlieb A, Stein GY, Oron Y, Ruppin E, Sharan R (2012). INDI: a computational framework for inferring drug interactions and their associated recommendations. Mol Syst Biol.

[12] Zhang W, Chen Y, Liu F, Luo F, Tian G, Li X (2017). Predicting potential drug-drug interactions by integrating chemical, biological, phenotypic and network data. BMC Bioinformatics.

[13] Vilar S, Uriarte E, Santana L, Lorberbaum T, Hripcsak G, Friedman C, Tatonetti NP (2014). Similarity-based modeling in large-scale prediction of drug-drug interactions. Nat Protoc.

[14] Cheng F, Zhao Z (2014). Machine learning-based prediction of drug-drug interactions by integrating drug phenotypic, therapeutic, chemical, and genomic properties. J Am Med Inform Assoc.

[15] Cheng W, Kasneci G, Graepel T, Stern D, Herbrich R (2011). Automated feature generation from structured knowledge. Proceedings of the 20th ACM International Conference on Information and Knowledge Management - CIKM ’11.

[16] Paulheim H, Fümkranz J (2012). Unsupervised generation of data mining features from linked open data. Proceedings of the 2nd International Conference on Web Intelligence, Mining and Semantics - WIMS ’12.

[17] Ristoski P, Bizer C, Paulheim H (2015). Mining the web of linked data with RapidMiner. Web Semant Sci Serv Agents World Wide Web.

[18] Su C, Tong J, Zhu Y, Cui P, Wang F. Network embedding in biomedical data science. Brief Bioinform. 2018;:117. 10.1093/bib/bby117. 10.1093/bib/bby117 30535359

[19] Grover A, Leskovec J (2016). node2vec: Scalable feature learning for networks. Proceedings of the 22nd ACM SIGKDD International Conference on Knowledge Discovery and Data Mining.

[20] Perozzi B, Al-Rfou R, Skiena S (2014). Deepwalk: Online learning of social representations. Proceedings of the 20th ACM SIGKDD International Conference on Knowledge Discovery and Data Mining.

[21] Celebi R, Yasar E, Uyar H, Gumus O, Dikenelli O, Dumontier M (2018). Evaluation of Knowledge Graph Embedding Approaches for Drug-Drug Interaction Prediction using Linked Open Data. International Conference on Semantic Web Applications and Tools for Healthcare and Life Sciences.

[22] Wishart DS, Feunang YD, Guo AC, Lo EJ, Marcu A, Grant JR, Sajed T, Johnson D, Li C, Sayeeda Z (2017). Drugbank 5.0: a major update to the drugbank database for 2018. Nucleic Acids Res.

[23] Law V, Knox C, Djoumbou Y, Jewison T, Guo AC, Liu Y, Maciejewski A, Arndt D, Wilson M, Neveu V (2013). Drugbank 4.0: shedding new light on drug metabolism. Nucleic Acids Res.

[24] Kanehisa M (2000). KEGG: Kyoto encyclopedia of genes and genomes. Nucleic Acids Res.

[25] Klein TE, Chang JT, Cho MK, Easton KL, Fergerson R, Hewett M, Lin Z, Liu Y, Liu S, Oliver DE, Rubin DL, Shafa F, Stuart JM, Altman RB (2001). Integrating genotype and phenotype information: an overview of the PharmGKB project. pharmacogenetics research network and knowledge base. Pharmacogenomics J.

[26] Sinha A, Cazabet R, Vaudaine R (2018). Systematic biases in link prediction: comparing heuristic and graph embedding based methods. International Conference on Complex Networks and Their Applications.

[27] Park Y, Marcotte EM (2012). Flaws in evaluation schemes for pair-input computational predictions. Nat Methods.

[28] Guney E (2018). Revisiting cross-validation of drug similarity based classifiers using paired data. Genomics Comput Biol.

[29] Shi J-Y, Li J-X, Gao K, Lei P, Yiu S-M (2017). Predicting combinative drug pairs towards realistic screening via integrating heterogeneous features. BMC Bioinformatics.

[30] Abdelaziz I, Fokoue A, Hassanzadeh O, Zhang P, Sadoghi M (2017). Large-scale structural and textual similarity-based mining of knowledge graph to predict drug–drug interactions. Web Semant Sci Serv Agents World Wide Web.

[31] Yu H, Mao K-T, Shi J-Y, Huang H, Chen Z, Dong K, Yiu S-M (2018). Predicting and understanding comprehensive drug-drug interactions via semi-nonnegative matrix factorization. BMC Syst Biol.

[32] Guney E (2017). Reproducible drug repurposing: When similarity does not suffice. PACIFIC SYMPOSIUM ON BIOCOMPUTING 2017.

[33] Shi J-Y, Huang H, Li J-X, Lei P, Zhang Y-N, Dong K, Yiu S-M (2018). Tmfuf: a triple matrix factorization-based unified framework for predicting comprehensive drug-drug interactions of new drugs. BMC Bioinformatics.

[34] Zhang Y, Zheng W, Lin H, Wang J, Yang Z, Dumontier M (2018). Drug-drug interaction extraction via hierarchical RNNs on sequence and shortest dependency paths. Bioinformatics.

[35] Noor A, Assiri A, Ayvaz S, Clark C, Dumontier M (2017). Drug-drug interaction discovery and demystification using semantic web technologies. J Am Med Inform Assoc.

[36] Cami A, Manzi S, Arnold A, Reis BY (2013). Pharmacointeraction network models predict unknown drug-drug interactions. PLoS ONE.

[37] Bobadilla J, Ortega F, Hernando A, Gutiérrez A (2013). Recommender systems survey. Knowl-Based Syst.

[38] Ferdousi R, Safdari R, Omidi Y (2017). Computational prediction of drug-drug interactions based on drugs functional similarities. J Biomed Inform.

[39] Shi J-Y, Mao K-T, Yu H, Yiu S-M (2019). Detecting drug communities and predicting comprehensive drug–drug interactions via balance regularized semi-nonnegative matrix factorization. J Cheminformatics.

[40] Celebi R, Mostafapour V, Yasar E, Gumus O, Dikenelli O (2015). Prediction of Drug-Drug interactions using pharmacological similarities of drugs. 2015 26th International Workshop on Database and Expert Systems Applications (DEXA).

[41] Zhang P, Wang F, Hu J, Sorrentino R (2015). Label propagation prediction of Drug-Drug interactions based on clinical side effects. Sci Rep.

[42] Li P, Huang C, Fu Y, Wang J, Wu Z, Ru J, Zheng C, Guo Z, Chen X, Zhou W, Zhang W, Li Y, Chen J, Lu A, Wang Y (2015). Large-scale exploration and analysis of drug combinations. Bioinformatics.

[43] Shi J-Y, Li J-X, Mao K-T, Cao J-B, Lei P, Lu H-M, Yiu S-M (2019). Predicting combinative drug pairs via multiple classifier system with positive samples only. Comput Methods Prog Biomed.

[44] Zhang W, Jing K, Huang F, Chen Y, Li B, Li J, Gong J (2019). Sflln: A sparse feature learning ensemble method with linear neighborhood regularization for predicting drug–drug interactions. Inf Sci.

[45] Luo H, Zhang P, Huang H, Huang J, Kao E, Shi L, He L, Yang L (2014). DDI-CPI, a server that predicts drug–drug interactions through implementing the chemical–protein interactome. Nucleic Acids Res.

[46] Hameed PN, Verspoor K, Kusljic S, Halgamuge S (2017). Positive-Unlabeled learning for inferring drug interactions based on heterogeneous attributes. BMC Bioinformatics.

[47] Ristoski P, Paulheim H (2016). RDF2Vec: RDF graph embeddings for data mining. Lecture Notes in Computer Science.

[48] Bordes A, Usunier N, Garcia-Duran A, Weston J, Yakhnenko O (2013). Translating embeddings for modeling multi-relational data. Advances in Neural Information Processing Systems.

[49] Ji G, He S, Xu L, Liu K, Zhao J (2015). Knowledge graph embedding via dynamic mapping matrix. Proceedings of the 53rd Annual Meeting of the Association for C7omputational Linguistics and the 7th International Joint Conference on Natural Language Processing (Volume 1: Long Papers), vol. 1.

[50] Bizer C, Heath T, Berners-Lee T (2009). Linked data - the story so far. Int J Semant Web Inf Syst.

[51] Berners-Lee T, Fielding R, Masinter L. Uniform resource identifiers (URI): Generic syntax. Technical report. 1998.

[52] Pan JZ (2009). Resource description framework. Handbook on Ontologies.

[53] Callahan A, Cruz-Toledo J, Ansell P, Dumontier M, Cimiano P, Corcho O, Presutti V, Hollink L, Rudolph S (2013). Bio2RDF release 2: Improved coverage, interoperability and provenance of life science linked data. The Semantic Web: Semantics and Big Data. Lecture Notes in Computer Science.

[54] Mikolov T, Sutskever I, Chen K, Corrado GS, Dean J (2013). Distributed representations of words and phrases and their compositionality. Advances in Neural Information Processing Systems.

[55] Ayvaz S, Horn J, Hassanzadeh O, Zhu Q, Stan J, Tatonetti NP, Vilar S, Brochhausen M, Samwald M, Rastegar-Mojarad M (2015). Toward a complete dataset of drug–drug interaction information from publicly available sources. J Biomed Inform.

[56] Crichton G, Guo Y, Pyysalo S, Korhonen A (2018). Neural networks for link prediction in realistic biomedical graphs: a multi-dimensional evaluation of graph embedding-based approaches. BMC Bioinformatics.

[57] Ryu JY, Kim HU, Lee SY (2018). Deep learning improves prediction of drug–drug and drug–food interactions. Proc Natl Acad Sci.

